# Clinical relevance of intraoperative blood loss in pancreatic surgery: a systematic review and meta-analysis to reappraise the impact on post operative pancreatic fistula

**DOI:** 10.1007/s00423-025-03902-6

**Published:** 2025-11-10

**Authors:** Giampaolo Perri, Danhui Heo, Rayner Peyser Cardoso, Swizel Ann Cardoso, Antonio Facciorusso, Riccardo Pellegrini, Domenico Bassi, Umberto Cillo, Giovanni Marchegiani

**Affiliations:** 1 Hepato Pancreato Biliary (HPB) and Liver Transplant Surgery, Department of Surgical, Oncological and Gastroenterological Sciences (DiSCOG), University of Padua, Via Giustiniani 2, Padova, 35128 Italy; 2 Doctoral School of Experimental and Preventive Medicine, Albert Szent-Györgyi Medical School, University of Szeged, Szeged, Hungary; 3 All India Institute of Medical Sciences, Jodhpur, India; 4 University Hospital of Birmingham NHS Foundation Trust, Birmingham, UK; 5 Gastroenterology Unit, Department of Experimental Medicine, Università del Salento, Lecce, 73100 Italy

**Keywords:** Intraoperative blood loss, Intraoperative bleeding, Pancreatic surgery, Pancreatic fistula, POPF

## Abstract

**Background:**

Postoperative pancreatic fistula (POPF) is the major complication following pancreatic surgery, significantly impacting patient outcomes. Intraoperative blood loss (IBL) represents a modifiable risk factor for POPF, but its actual clinical relevance is not clearly defined. This study explores the available literature to reappraise the association of IBL and the development of POPF.

**Methods:**

A systematic review and meta-analysis of original studies published between January 2006 and August 2025, reporting IBL in patients undergoing pancreatic resections and its association with POPF were performed. Studies that used the International Study Group on Pancreatic Surgery (ISGPS) or the International Study Group on Pancreatic Fistula Definition (ISGPF) definitions for POPF were included. Qualitative synthesis included all eligible studies; quantitative meta-analysis was conducted for studies reporting IBL in both POPF and no-POPF groups.

**Results:**

A total of 26 studies were included in the qualitative review and 12 in the meta-analysis. Among 13,108 patients who underwent pancreatic resections, the overall POPF rate was 20%. IBL was identified as an independent risk-factor of POPF in 17 studies. The meta-analysis, which included 10,008 patients, showed that IBL was significantly higher in the POPF group compared to the no-POPF group [Mean difference (MD): 112.46 ml (30.39, 194.53), *p* = 0.01].

**Conclusions:**

IBL is an independent predictor of POPF. Intraoperative measures to minimize its occurrence and magnitude are key to ameliorate the outcomes of pancreas surgery.

**Supplementary Information:**

The online version contains supplementary material available at 10.1007/s00423-025-03902-6.

## Introduction

Despite advances in surgical techniques, perioperative care, and anesthesia have significantly improved the outcomes, pancreatic surgery still is characterized by high rates of postoperative mortality and morbidity. Among these complications, postoperative pancreatic fistula (POPF) represents the most frequent and concerning complication after pancreatic surgery [1–3]. POPF is a dangerous complication that can trigger serious secondary problems. The leaked pancreatic fluid contains proteolytic enzymes that damage surrounding tissues and erode blood vessels, causing severe hemorrhage requiring emergency intervention [4, 5]. POPF also creates a persistent source of intra-abdominal infection, leading to abscess formation and sepsis [4, 6]. POPF management is complex and typically requires prolonged drainage, percutaneous interventions, and occasionally completion pancreatectomy, resulting in extended hospital stays and substantially elevated healthcare costs [7, 8]. Moreover, POPF has frequently been associated with delays in initiating adjuvant chemotherapy, or even the inability to receive it, which may adversely affect long-term oncologic outcomes [9].

Intraoperative blood loss (IBL) is a key factor influencing postoperative outcomes, particularly in hepato-pancreato-biliary (HPB) surgery, where it has been frequently associated with adverse perioperative outcomes [10–13]. In pancreatic surgery, high IBL may contribute to relative ischemia of the pancreatic remnant, potentially impairing healing of the pancreatico-enteric anastomosis [14, 15]. Furthermore, aggressive fluid resuscitation in response to blood loss can result in tissue edema [16, 17], while perioperative blood transfusions may induce immunosuppression [18, 19], both of which can further compromise anastomotic integrity.

However, the relationship between IBL and POPF remains controversial. While excessive IBL is often associated with increased postoperative morbidity and mortality, its direct impact on the development of POPF is not fully elucidated.

Therefore, this study aims to systematically review the existing literature and perform a meta-analysis to evaluate the clinical impact of IBL in pancreatic surgery, with particular focus on its association with the development of POPF.

## Methods

### Study design

A systematic literature search was performed according to the Preferred Reporting Items for Systematic Reviews and Meta-analysis (PRISMA) guidelines [20]. A protocol has been registered in PROSPERO (https://www.crd.york.ac.uk/PROSPERO/view/CRD42025649406). The study was initially designed to include HPB surgeries, encompassing both liver and pancreatic surgeries. However, during the full-text assessment showed that there were insufficient data on liver surgery outcomes to perform a meta-analysis. As a result, the scope of the study was narrowed to focus exclusively on pancreatic surgery. The flowchart and subsequent analyses were revised accordingly to reflect this refined focus.

The PubMed database was searched for full-text, English-written studies published before September 1, 2025. The search string is available as supplementary data (Supplementary Fig. 1). Although liver surgery was initially included in the search strategy, these studies were subsequently excluded as insufficient data were available to perform a meta-analysis on liver surgery outcomes. Studies were included if they applied either the International Study Group of Pancreatic Fistula (ISGPF) 2005 definition or the updated International Study Group of Pancreatic Surgery (ISGPS) 2016 definition for clinically relevant POPF (CR-POPF) [21, 22]. The primary outcome of interest was the occurrence of POPF. Studies were eligible if they reported IBL stratified by POPF versus no POPF, or by CR-POPF versus no CR-POPF. In the ISGPS 2016 definition, CR-POPF corresponds to grades B and C. For studies using the ISGPF 2005 classification, POPF grade B and C were also considered as CR-POPF. All studies published before January 1, 2006, case reports or descriptions of surgical techniques, series with < 20 patients, studies with IBL cited only in the abstract or not referring to pancreatic resections, duplicates, editorials, systematic reviews/meta-analyses, studies without free-access records, and animal studies were excluded from the systematic review.

During the screening at least two researchers (A.M., G.P., D.H., and R.P.C.) independently screened all the identified studies. After a first screening based on the title and abstract for assessing full-text eligibility, two researchers independently performed the full-text assessment (A.M., G.P., D.H., and R.P.C.). A final consensus was reached after discussion with a senior researcher (G.M.) in cases of any disagreement. Statistical analysis was performed by two authors (D.H., A.F.) including a second senior researcher (A.F.).

### Statistical analysis

The risk of bias was determined using the ROBINS-I tool for cohort studies. The methodological quality of the included studies was evaluated independently by two authors (S.A.C and R.P.C) (Supplementary Fig. 2). The meta-analysis was conducted through a random-effects model using Restricted Maximum Likelihood Estimation (REML) to calculate mean difference (MD) and 95% confidence intervals (CI) between POPF vs. no-POPF in pancreatic surgery patients. If the outcomes were reported in medians with interquartile range (IQR) or 95% CI or range the mean and standard deviation values were calculated from the statistical estimations in accordance with the Cochrane manual and Wan et al. [23, 24]. Heterogeneity between study-specific estimates was estimated using the inconsistency index (I^2^), and cutoffs of < 30%, 30%–59%, 60%–75% and >75% suggested low, moderate, substantial, and considerable heterogeneity, respectively [25]. Publication bias was assessed qualitatively using funnel plot asymmetry (Supplementary Fig. 3). Additionally, the certainty of evidence for outcomes related to POPF was assessed using the updated Grading of Recommendations Assessment, Development, and Evaluation (GRADE) approach (Supplementary Fig. 4). All analysis was performed using RevMan 5.3 software (the Cochrane Collaboration).

## Sensitivity analysis

We conducted a leave-one-out sensitivity analysis to evaluate the influence of individual studies on the pooled estimates. In this procedure, each study was excluded in turn and the meta-analysis was repeated to assess any changes in effect size or heterogeneity. Outcomes that lost or gained statistical significance after exclusion were noted, whereas results that remained unchanged were considered stable.

## Results

**Fig. 1:**
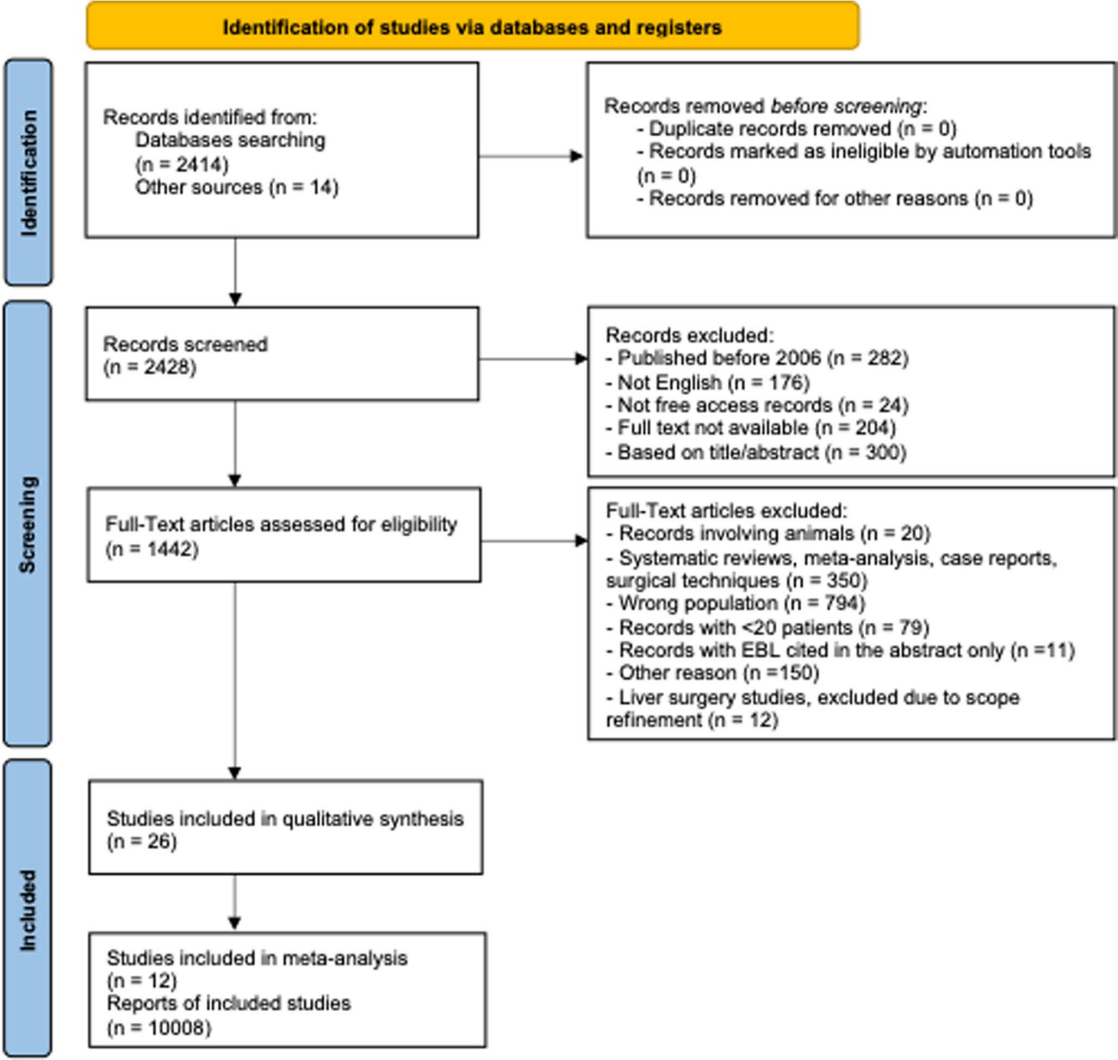
The PRISMA study selection

The PRISMA study selection flowchart is shown in Fig. 1. The systematic search identified 2414 studies, and an additional 14 were added through cross-checking references. A total of 1442 studies were screened for full-text eligibility. Of these, 26 studies met the criteria for full-text review and were included in the qualitative synthesis [14, 26–50]. Among them, only 12 were eligible for statistical analysis and were included in the quantitative synthesis [14, 28, 31, 33, 34, 36, 41, 44, 47–50].

The characteristics of the 26 studies on pancreatic surgery are summarized in Table 1. Twenty (77%) focused only on pancreatoduodenectomy (PD), 5 (19%) only on distal pancreatectomy (DP), and 1 (4%) on both. Twelve studies (46%) used the updated ISGPS definition of POPF [22], and 14 (54%) referred to CR-POPF according to the previous ISGPF definition [21]. Among the 13,108 patients included, 20% developed POPF. The lowest rate of POPF was reported by Malleo et al. [42] at 13%, while Dai et al. [14] had the highest rate at 57.8%. Seventeen studies evaluated blood loss as an independent risk factor for POPF by multivariate analysis [14, 16, 28, 34, 41–48, 50–54]. Of these, 12 were included in the qualitative synthesis, while 5 additional studies were analyzed separately because they were excluded from the qualitative analysis due to patient duplication and other limitations.

**Table 1:** Characteristics of the included studies

Characteristics			* N* (%)
Year			
	2010–2015	6 (23)	
	2016–2025	20 (77)	
Study Type			
	Retrospective	23 (88)	
	Prospective	3 (12)	
**PANCREATIC SURGERY** (*N* = 26)			
Resection Type			
	PD	20 (77)	
	DP	5 (19)	
	Both	1 (4)	
POPF definition			
	ISGPF	14 (54)	
	ISGPS	12 (46)	
**PATIENTS** (*N* = 13108)			
CR-POPF			
	Yes	2633 (20)	
	No	10,385 (80)	

## Meta-analysis

### Risk of bias assessment

The risk of bias assessment using the ROBINS-I tool is shown in Supplementary Fig. 2. Among the 12 studies included in the meta-analysis, nine were assessed as having a low or moderate risk of bias, while three had a serious risk, mainly due to residual confounding and deviations from intended interventions. Overall, most studies demonstrated a low to moderate risk of bias.

## Association between intraoperative blood loss and POPF

**Fig. 2:**
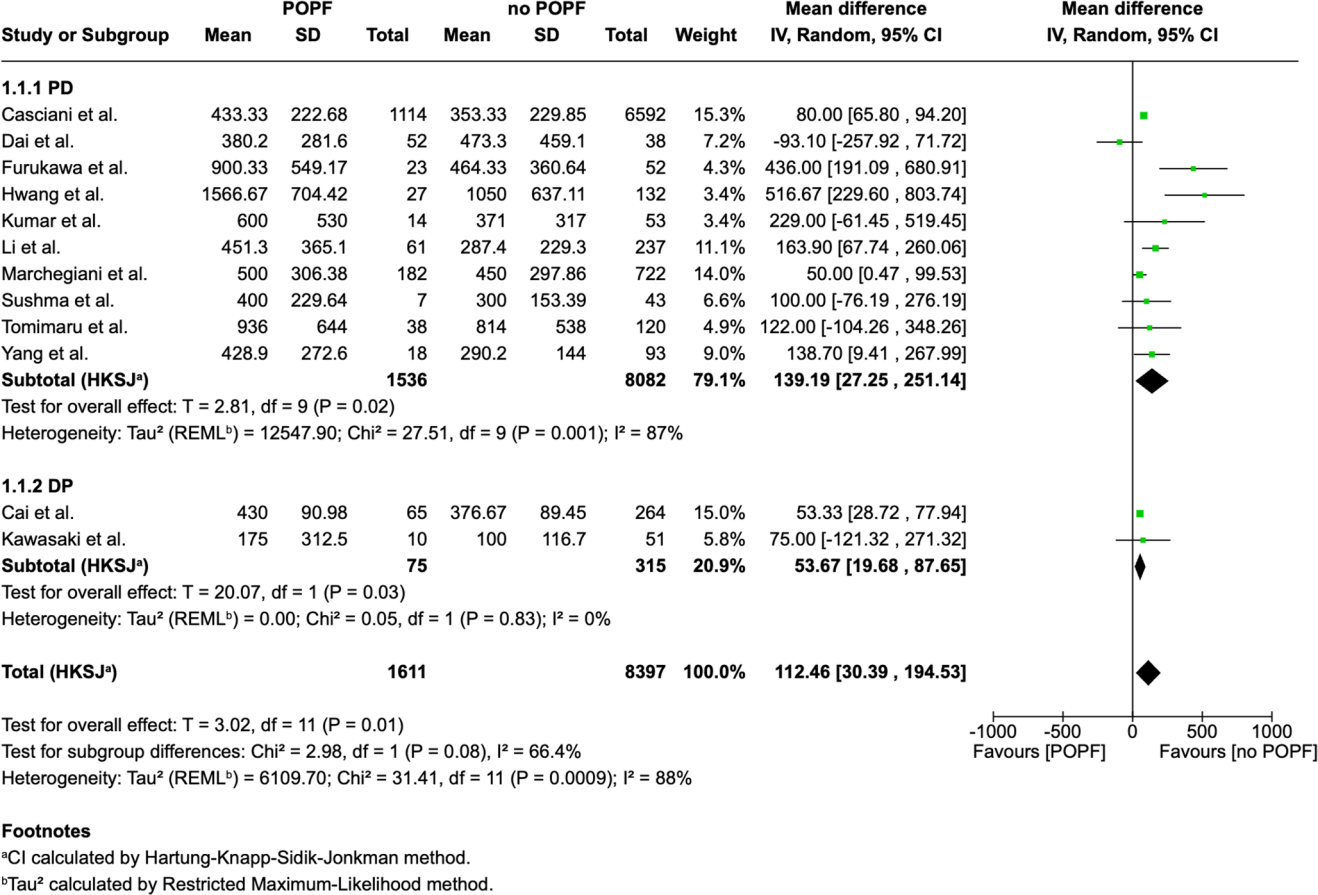
The forest plot

The characteristics of the 12 studies that included in the quantitative synthesis are summarized in Table 2. Among the 12 studies included in the meta-analysis [14, 28, 31, 33, 34, 36, 41, 44, 47–50], patients underwent PD or DP. Five studies (42%) used the CR-POPF ISGPF definition and the other Seven (58%) used the ISGPS updated definition. In total, 10,008 patients were analyzed, of whom 1611 developed POPF (overall POPF rate of 16%). The lowest median IBL in POPF patients in the PD group was 380 ml, reported by Dai et al. [49]and lowest median IBL in POPF in all groups including PD and DP was 175 ml, reported by Kawasaki et al. [36]. After statistical analysis, a significant mean difference of 112.46 ml (30.39, 194.53; *p* = 0.01) between POPF group and no-POPF group was found, with higher intraoperative blood loss associated with POPF development (Fig. 2).

**Table 2:** Characteristics of included studies in the meta–analysis

Study	Design	Country	Study period	POPF definition	Group	Patients number	Age, y	Sex (Female)	Texture (Soft)	Duct size, mm	Pathology
Casciani et al.^*^[47]	Retrospective	International	2003–2020	ISGPS	POPF	14.5% (*N*=1114)	N/A	N/A	85.6% (*N*=798)	≥ 3: 56.8% (*N*=529) < 3: 43.2% (*N*=403)	(Pancreatic cancer or pancreatitis): 32.1% (*N*=299)
					no POPF	85.5% (*N*=6592)	N/A	N/A	48.0% (*N*=2335)	≥ 3: 76.6% (*N*=3723) < 3: 23.4% (*N*=1140)	(Pancreatic cancer or pancreatitis): 43.7% (*N*=2123)
Cai et al. [50]	Restrospective	China	2019–2023	ISGPS	POPF	19.8% (*N*=65)	61.4 ± 4.5^§^	46.2% (*N*=30)	73.9% (*N*=48)	2.0 (1.3–2.6)^†^	N/A
					no POPF	80.2% (*N*=264)	65.7 ± 3.9^§^	40.9% (*N*=108)	29.2% (*N*=77)	2.3 (1.5–2.9)^†^	N/A
Dai et al. [49]	Retrospective	China	2014–2023	ISGPS	POPF	57.8% (*N*=52)	65.8 ± 10.9^‡^	11.5% (*N*=6)	64.1% (*N*=25)	3.0 ± 0.1^‡^	(Malignant): 84.6% (*N*=44)
					no POPF	42.2% (*N*=38)	63.1 ± 9.9^‡^	26.3% (*N*=10)	27.3% (*N*=6)	3.6 ± 0.2^‡^	(Malignant): 92.1% (*N*=35)
Furukawa et al. [14]	Retrospective	Japan	2014–2020	ISGPF	POPF	30.7% (*N*=23)	71 (62–74)^†^	21.7% (*N*=5)	87.0% (*N*=20)	2 (2–4)^†^	(Malignant): 13.0% (*N*=3)
					no POPF	69.3% (*N*=52)	68 (63–77)^†^	36.5% (*N*=19)	55.8% (*N*=29)	2 (2–2)^†^	(Malignant): 44.2% (*N*=3)
Hwang et al. [44]	Retrospective	Korea	2002–2008	ISGPF	POPF	17.0% (*N*=27)	62.9 ± 11.2^§^	N/A	N/A	3 (2–3)^†^	N/A
					no POPF	83.0% (*N*=132)	59.6 ± 12.0^§^	N/A	N/A	3 (2–5)^†^	N/A
Kawasaki et al. [36]	Retrospective	Korea	2006–2016	ISGPF	POPF	16.4% (*N*=10)	36 (24–80)	70.0% (*N*=7)	70.0% (*N*=7)	N/A	N/A
					no POPF	83.6% (*N*=51)	52 (21–86)	66.7% (*N*=34)	81.6% (*N*=40)	N/A	N/A
Kumar et al. [28]	Retrospective	India	2015–2017	ISGPF	POPF	20.9% (*N*=14)	48.2 ± 12.1^‡^	57.1% (*N*=8)	71.4% (*N*=10)	3.5 ± 2.6^‡^	(Malignant): 100% (*N*=14)
					no POPF	79.1% (*N*=53)	48.2 ± 11.3^‡^	39.6% (*N*=21)	46.5% (*N*=20)	4.3 ± 2.9^‡^	(Malignant): 100% (*N*=53)
Li et al. [34]	Retrospective	China	2011–2016	ISGPS	POPF	20.5% (*N*=61)	62.2 ± 9.4^§^	32.8% (*N*=20)	82.0% (*N*=50)	2.6 ± 0.7^§^	(Malignant): 91.8% (*N*=56)
					no POPF	79.5% (*N*=237)	61.8 ± 10.1^§^	43.0% (*N*=102)	33.8% (*N*=80)	3.2 ± 1.0^§^	(Malignant): 94.5% (*N*=224)
Marchegiani et al. [48]	Prospective	Italy	2016–2021	ISGPS	POPF	20.1% (*N*=182)	N/A	N/A	N/A	N/A	N/A
					no POPF	79.9% (*N*=722)	N/A	N/A	N/A	N/A	N/A
Sushma et al. [31]	Prospective	India	2019–2020	ISGPS	POPF	14.0% (*N*=7)	48 ± 13^§^	N/A	85.7% (*N*=6)	4 (3.0-4.5)^†^	N/A
					no POPF	86.0% (*N*=43)	54 ± 15^§^	N/A	32.6% (*N*=14)	4 (3.0-7.0)^†^	N/A
Tomimaru et al. [33]	Retrospective	Japan	2009–2017	ISGPS	POPF	24.1% (*N*=38)	70 ± 7^§^	26.3% (*N*=10)	97.4% (*N*=37)	2.8 ± 1.1^§^	(Malignant): 57.9% (*N*=22)
					no POPF	75.9% (*N*=120)	70 ± 10^§^	45.8% (*N*=55)	43.3% (*N*=52)	4.5 ± 2.0^§^	(Malignant): 77.5% (*N*=93)
Yang [41]	Retrospective	China	2010–2013	ISGPF	POPF	16.2% (*N*=18)	56.6 ± 9.8^§^	44.4% (*N*=8)	83.3% (*N*=15)	≥ 3: 88.9% (*N*=16) < 3: 11.1% (*N*=2)	(Malignant): 83.3% (*N*=15)
					no POPF	83.8% (*N*=93)	56.4 ± 10.3^§^	51.6% (*N*=48)	75.3% (*N*=70)	≥ 3: 61.3% (*N*=57) < 3: 38.7% (*N*=36)	(Malignant): 87.1% (*N*=81)

*This study, total population (7706) and patient characteristic population (5795) are different. † Median, (IQR) is used. ‡ Median, CI 95% is used. § Mean, SD is used. Duct size refers to the main pancreatic duct (MPD). *POPF* postoperative pancreatic fistula; *ISGPS* international study group on pancreatic surgery; *ISGPF* international study group of pancreatic fistula

## Subgroup analysis

Subgroup analysis was conducted to separate PD and DP groups in order to evaluate the impact of IBL on POPF within each surgical type. Of the 12 studies, ten were included in the PD subgroup and two in the DP subgroup. Both subgroups demonstrated a statistically significant association between IBL and POPF. The PD subgroup showed a mean difference of 139.19 mL (27.25, 251.14; *p* = 0.02), and the DP subgroup showed a mean difference of 53.67 mL (19.68, 87.65; *p* = 0.03). While the PD subgroup continued to show high heterogeneity, the DP subgroup demonstrated markedly lower heterogeneity (I² = 0%).

### Sensitivity analysis

A leave-one-out sensitivity analysis was performed to examine the impact of individual studies on the pooled estimates. Sequential removal of each study did not alter the statistical significance of the results, either in the PD subgroup or in the overall analysis. Across the leave-one-out tests, heterogeneity varied between 70 and 91% in the PD subgroup and 33–93% in the overall analysis. Notably, exclusion of Furukawa et al. or Hwang et al. led to a marked decrease in heterogeneity in both the PD subgroup and the overall analysis.

### GRADE assessment

Supplementary Fig. 4 summarizes the certainty of evidence for the relationship between IBL and POPF. The overall estimate (12 studies; MD 112.46 mL higher, 95% CI 30.39–194.53) was graded as very low because of limitations in study quality, substantial heterogeneity, and indications of publication bias. The PD subgroup (10 studies; MD 112.46 mL higher, 95% CI 30.39–194.53) likewise showed very low certainty, mainly due to inconsistency and possible publication bias. In contrast, the DP subgroup (2 studies; MD 53.67 mL higher, 95% CI 19.68–87.65) was rated as low certainty, mainly due to the observational study design, but without major concerns regarding risk of bias or inconsistency.

## Discussion

As there is already solid evidence that POPF is the major cause of postoperative severe morbidity and mortality after pancreatic surgery [1, 2, 7], the present analysis found that IBL is a key intraoperative risk factor for its development [16, 47]. Higher IBL may contribute to POPF with different pathways. First, high IBL may cause relative ischemia of pancreatic remnant, impairing the healing of the pancreatic anastomosis [15, 16, 18, 55, 56]. Relative ischemia of pancreatic remnant also plays a fundamental role in the etiology of postoperative serum hyperamylasemia (POH) and post-pancreatectomy acute pancreatitis (PPAP) [57, 58], that demonstrated relevant implication on POPF development [59, 60]. Additionally, IBL related transfusions might lead to immunosuppression, tissue edema secondary to aggressive volume replacement, and the inflammatory response triggered by ischemia-reperfusion injury [14–16, 18, 19, 51].

Moreover, maintaining a bloodless surgical field reduces the risk of injuring important structures, particularly biliary ducts, and consequently prevents postoperative biliary complications. Another advantage of keeping low IBL is the ability to visualize all bleeding sites during parenchymal resection and thus perfect hemostasis before closure. Blood is also an excellent culture medium, so minimizing IBL may contribute to a lower rate of infectious complications.

The relevance of IBL in pancreatic surgery was confirmed in 17 different studies which identified it as an independent risk factor for POPF [14, 16, 28, 34, 41–48, 50–54] and IBL is consequently one of the four factors included in the Fistula Risk Score [16]. Callery et al. [16] found that IBL >1000 ml was the most significant factor predisposing patients to POPF [OR: 5.60 (1.65, 18.98), *p* = 0.006]. Additionally, an increased risk of pancreatic fistula was observed with higher intraoperative blood loss, particularly above the 400 ml [47, 52].

Notably, high blood loss contributes to POPF development both in negligible and high-risk patients. Trudeau et al. [51] found that in patients with negligible endogenous risk (≥ 5 mm duct, protective pathology and firm pancreas), EBL >400 ml was associated with a 5-fold increase in POPF occurrence. Moreover, in high-risk patients (small duct, high risk pathology and soft gland) blood loss is the only modifiable risk factor, so minimizing IBL is even more important, and it should be considered among the strategies to mitigate the risk of POPF development [51, 52]. Consistently with these findings, the meta-analysis confirmed these results. Among 8 studies analyzed, with 8624 patients included and overall POPF rate of 15%, significantly higher IBL was found in patients who developed POPF compared to those who did not.

Multiple risk factors for major intraoperative bleeding during pancreatic surgery have been deeply analyzed and identified in several studies. Preoperatively, male sex, BMI ≥ 25 kg/m^2^, biliary drainage, anticoagulation or antiplatelet treatment, liver function, and neo-adjuvant chemotherapy were found to be associated with high IBL [47, 53, 61]. Intraoperatively, locally advanced disease, vascular and multiorgan resection, and resulting longer operative duration increased the risk of extensive bleeding [53, 62]. Regarding surgical approach, minimally invasive techniques demonstrated to reduce IBL [29, 51, 53]. Finally considering pancreas-specific factors, soft gland texture, non-pancreatic cancer or pancreatitis disease pathology were positively associated with low IBL [47, 53].

To reduce intraoperative bleeding, the anticipatory dissection approach should be applied, with proper exposure of the field and vessels control, as well as accurate hemostasis using all available devices (suture ligation, cautery, staplers) [51, 53, 63]. Some of them have proven to provide a more stable hemostatic seal and a reduction in operative time [64, 65]. Moreover, specifically in pancreatic surgery, the artery first approach might also provide not only better oncologic outcomes but also reduced intraoperative bleeding [66].

It is important to note that in this meta-analysis, we attempted to perform a subgroup analysis of the association between IBL and POPF by considering potential risk factors such as pancreatic duct size and gland texture. However, due to inconsistencies in reporting and the lack of stratification in the included studies, conducting a detailed, separate analysis based on these factors was not achievable.

The present review has several limitations. First, most of the included studies were retrospective and therefore characterized by the inherent risk of heterogeneity. Furthermore, the meta-analysis examining the relationship between IBL and POPF, the funnel plot reveals asymmetry, suggesting potential publication bias with possible overestimation of the association. This highlights the need for more comprehensive reporting of both positive and negative results in future studies of IBL and POPF.

## Conclusion

This review confirmed that IBL is an important risk factor for POPF, and it must be considered as a quality indicator in pancreatic surgery. Despite this pronounced clinical relevance in contributing to postoperative outcomes, not all studies in the literature reported median IBLs in complication-group compared with standard postoperative course. In the future, in addition to further efforts to minimize blood loss, it will be necessary to emphasize the need for IBL reporting in all HPB papers, and identify a standardized, universal definition of IBL to allow accurate comparisons across studies and increase the clinical utility of existing risk scores.

## Supplementary Information

Below is the link to the electronic supplementary material.

Supplementary Material 1

Supplementary Material 2

## Data Availability

No datasets were generated or analysed during the current study.

## References

[1] Vollmer CM Jr. et al (2012) A root-cause analysis of mortality following major pancreatectomy. J Gastrointest Surg 16(1):89–102 discussion 102-3 22065319 10.1007/s11605-011-1753-x

[2] Malleo G et al (2014) Diagnosis and management of postoperative pancreatic fistula. Langenbecks Arch Surg 399(7):801–810 25173359 10.1007/s00423-014-1242-2

[3] Marchegiani G, Bassi C (2021) Prevention, prediction, and mitigation of postoperative pancreatic fistula. Br J Surg 108(6):602–604 33942063 10.1093/bjs/znab125

[4] Crippa S et al (2007) Anastomotic leakage in pancreatic surgery. HPB 9(1):8–15 18333107 10.1080/13651820600641357 PMC2020778

[5] Marchetti A (2025) Reoperation for pancreatic fistula: a systematic review of completion pancreatectomy vs. pancreas-preserving-procedures and outcomes. HPB (Oxford) 27(2):240–249 39658409 10.1016/j.hpb.2024.11.006

[6] Marchegiani G et al (2019) Current definition of and controversial issues regarding postoperative pancreatic fistulas. Gut Liver 13(2):149–153 30419630 10.5009/gnl18229 PMC6430431

[7] Williamsson C et al (2017) Postoperative pancreatic fistula-impact on outcome, hospital cost and effects of centralization. HPB (Oxford) 19(5):436–442 28161218 10.1016/j.hpb.2017.01.004

[8] Pulvirenti A et al (2018) Clinical implications of the 2016 international study group on pancreatic surgery definition and grading of postoperative pancreatic fistula on 775 consecutive pancreatic resections. Ann Surg 268(6):1069–1075 28678062 10.1097/SLA.0000000000002362

[9] Bonaroti JW et al (2021) Impact of postoperative pancreatic fistula on long-term oncologic outcomes after pancreatic resection. HPB (Oxford) 23(8):1269–1276 33526357 10.1016/j.hpb.2020.12.010 PMC8282784

[10] Pratt W, Callery MP, Vollmer CM Jr (2008) Optimal surgical performance attenuates physiologic risk in high-acuity operations. J Am Coll Surg 207(5):717–730 10.1016/j.jamcollsurg.2008.06.319 18954785

[11] Ejaz A et al (2014) Variation in triggers and use of perioperative blood transfusion in major Gastrointestinal surgery. Br J Surg 101(11):1424–1433 25091410 10.1002/bjs.9617

[12] Dixon E et al (2009) Blood loss in surgical oncology: neglected quality indicator? J Surg Oncol 99(8):508–512 19466741 10.1002/jso.21187

[13] Perri G et al (2024) Estimation of intraoperative blood loss in hepatopancreatobiliary surgery: a Delphi consensus process of the European-African Hepato-Pancreato-Biliary association (E-AHPBA). Br J Surg 111(10) 10.1093/bjs/znae256 PMC11977757 39387472

[14] Furukawa K et al (2021) Intraoperative amylase level of pancreatic juice as a simple predictor of pancreatic fistula after pancreaticoduodenectomy. Pancreatology 21(1):299–305 33214083 10.1016/j.pan.2020.10.048

[15] Strasberg SM, McNevin MS (1998) Results of a technique of pancreaticojejunostomy that optimizes blood supply to the pancreas. J Am Coll Surg 187(6):591–596 9849731 10.1016/s1072-7515(98)00243-9

[16] Callery MP et al (2013) A prospectively validated clinical risk score accurately predicts pancreatic fistula after pancreatoduodenectomy. J Am Coll Surg 216(1):1–14 23122535 10.1016/j.jamcollsurg.2012.09.002

[17] Andrianello S et al (2018) Clinical implications of intraoperative fluid therapy in pancreatic surgery. J Gastrointest Surg 22(12):2072–2079 30066067 10.1007/s11605-018-3887-6

[18] Thompson SK, Chang EY, Jobe BA (2006) Clinical review: healing in gastrointestinal anastomoses, part I. Microsurgery 26(3):131–136 16518804 10.1002/micr.20197

[19] Rohde JM et al (2014) Health care-associated infection after red blood cell transfusion: a systematic review and meta-analysis. JAMA 311(13):1317–1326 24691607 10.1001/jama.2014.2726 PMC4289152

[20] Moher D et al (2009) Preferred reporting items for systematic reviews and meta-analyses: the PRISMA statement. PLoS Med 6(7):e1000097 19621072 10.1371/journal.pmed.1000097 PMC2707599

[21] Bassi C et al (2005) Postoperative pancreatic fistula: an international study group (ISGPF) definition. Surgery 138(1):8–13 16003309 10.1016/j.surg.2005.05.001

[22] Bassi C et al (2017) The 2016 update of the international study group (ISGPS) definition and grading of postoperative pancreatic fistula: 11 years after. Surgery 161(3):584–591 28040257 10.1016/j.surg.2016.11.014

[23] Cumpston M et al (2019) Updated guidance for trusted systematic reviews: a new edition of the Cochrane handbook for systematic reviews of interventions. Cochrane Database Syst Rev 10(10): Ed000142 10.1002/14651858.ED000142 PMC10284251 31643080

[24] Wan X et al (2014) Estimating the sample mean and standard deviation from the sample size, median, range and/or interquartile range. BMC Med Res Methodol 14:135 25524443 10.1186/1471-2288-14-135 PMC4383202

[25] Higgins JP (2003) Measuring inconsistency in meta-analyses. BMJ 327(7414):557–560 12958120 10.1136/bmj.327.7414.557 PMC192859

[26] Saito R et al (2021) Exposure to blood components and inflammation contribute to pancreatic cancer progression. Ann Surg Oncol 28(13):8263–8272 34101067 10.1245/s10434-021-10250-4

[27] Iseki M (2021) A deep pancreas is a novel predictor of pancreatic fistula after pancreaticoduodenectomy in patients with a nondilated main pancreatic duct. Surgery 169(6):1471–1479 33390302 10.1016/j.surg.2020.11.033

[28] Kumar S et al (2021) Predictors and outcomes of pancreatic fistula following pancreaticoduodenectomy: a dual center experience. Indian J Surg Oncol 12(1):22–30 33814828 10.1007/s13193-020-01195-3 PMC7960792

[29] Huang L et al (2020) The effectiveness, risks and improvement of laparoscopic pancreaticoduodenectomy during the learning curve: a propensity score-matched analysis. Gland Surg 9(4):985–999 32953607 10.21037/gs-20-98 PMC7475375

[30] Jin KM et al (2020) The individualized selection of pancreaticoenteric anastomosis in pancreaticoduodenectomy. BMC Surg 20(1):140 32571289 10.1186/s12893-020-00791-y PMC7310108

[31] Sushma N et al (2020) Role of ultrasound shear wave elastography in preoperative prediction of pancreatic fistula after pancreaticoduodenectomy. Pancreatology 20(8):1764–1769 33139201 10.1016/j.pan.2020.10.047

[32] Yang F et al (2019) Drain contamination after distal pancreatectomy: incidence, risk Factors, and association with postoperative pancreatic fistula. J Gastrointest Surg 23(12):2449–2458 30815778 10.1007/s11605-019-04155-7

[33] Tomimaru Y et al (2019) Factors affecting healing time of postoperative pancreatic fistula in patients undergoing pancreaticoduodenectomy. Mol Clin Oncol 10(4):435–440 30931113 10.3892/mco.2019.1812 PMC6425506

[34] Li Y et al (2019) Novel risk scoring system for prediction of pancreatic fistula after pancreaticoduodenectomy. World J Gastroenterol 25(21):2650–2664 31210716 10.3748/wjg.v25.i21.2650 PMC6558436

[35] Winer LK et al (2018) Perioperative net fluid balance predicts pancreatic fistula after pancreaticoduodenectomy. J Gastrointest Surg 22(10):1743–1751 29869090 10.1007/s11605-018-3813-y

[36] Kawasaki Y et al (2018) Improved perioperative outcomes of laparoscopic distal pancreatosplenectomy: modified Lasso technique. ANZ J Surg 88(9):886–890 29266719 10.1111/ans.14351

[37] Shamali A (2017) Impact of obesity on short and long term results following a pancreatico-duodenectomy. Int J Surg 42:191–196 28461146 10.1016/j.ijsu.2017.04.058

[38] Han IW (2017) Excess intraoperative fluid volume administration is associated with pancreatic fistula after pancreaticoduodenectomy: a retrospective multicenter study. Medicine 96(22):e6893 28562540 10.1097/MD.0000000000006893 PMC5459705

[39] Hu BY et al (2016) Risk factors for postoperative pancreatic fistula: analysis of 539 successive cases of pancreaticoduodenectomy. World J Gastroenterol 22(34):7797–7805 27678363 10.3748/wjg.v22.i34.7797 PMC5016380

[40] Wang G et al (2016) External versus internal pancreatic duct drainage for the early efficacy after pancreaticoduodenectomy: A retrospectively comparative study. J Invest Surg 29(4):226–233 26822175 10.3109/08941939.2015.1105327

[41] Yang H (2015) Application of air insufflation to prevent clinical pancreatic fistula after pancreaticoduodenectomy. World J Gastroenterol 21(6):1872–1879 25684954 10.3748/wjg.v21.i6.1872 PMC4323465

[42] Malleo G et al (2014) Assessment of a complication risk score and study of complication profile in laparoscopic distal pancreatectomy. J Gastrointest Surg 18(11):2009–2015 25238815 10.1007/s11605-014-2651-9

[43] Hashimoto Y, Traverso LW (2012) After distal pancreatectomy pancreatic leakage from the stump of the pancreas may be due to drain failure or pancreatic ductal back pressure. J Gastrointest Surg 16(5):993–1003 22392088 10.1007/s11605-012-1849-y

[44] Hwang HK et al (2011) The impact of body mass index on pancreatic fistula after pancreaticoduodenectomy in Asian patients on the basis of Asia-Pacific perspective of body mass index. Jop 12(6):586–592 22072248

[45] Kawai M et al (2011) Predictive risk factors for clinically relevant pancreatic fistula analyzed in 1,239 patients with pancreaticoduodenectomy: multicenter data collection as a project study of pancreatic surgery by the Japanese society of hepato-biliary-pancreatic surgery. J Hepatobiliary Pancreat Sci 18(4):601–608 21491103 10.1007/s00534-011-0373-x

[46] Akamatsu N et al (2010) Risk factors for postoperative pancreatic fistula after pancreaticoduodenectomy: the significance of the ratio of the main pancreatic duct to the pancreas body as a predictor of leakage. J Hepato-Biliary-Pancreat Sci 17(3):322–328 10.1007/s00534-009-0248-6 20464562

[47] Casciani F et al (2021) The effect of high intraoperative blood loss on pancreatic fistula development after pancreatoduodenectomy: an international, multi-institutional propensity score matched analysis. Surgery 170(4):1195–1204 33931208 10.1016/j.surg.2021.03.044

[48] Marchegiani G et al (2023) Blood loss predicts pancreas-specific complications only in high-risk patients: results of a prospective and systematic blood loss estimation during pancreatoduodenectomy. Br J Surg 110(12):1632–1636 37406083 10.1093/bjs/znad207

[49] Dai Y et al (2025) Differences in grade C postpancreatectomy hemorrhage with or without clinically relevant pancreatic fistula after pancreaticoduodenectomy: a retrospective cohort study. Surgery 183:109355 40245597 10.1016/j.surg.2025.109355

[50] Cai W et al (2025) Baseline body composition and 3D-extracellular volume fraction for predicting pancreatic fistula after distal pancreatectomy in pancreatic body and/or tail adenocarcinoma. Acad Radiol 32(4):2027–2040 39537519 10.1016/j.acra.2024.10.010

[51] Trudeau MT et al (2022) The influence of intraoperative blood loss on fistula development following pancreatoduodenectomy. Ann Surg 276(5):e527–e535 33201132 10.1097/SLA.0000000000004549

[52] Casciani F et al (2021) Surgeon experience contributes to improved outcomes in pancreatoduodenectomies at high risk for fistula development. Surgery 169(4):708–720 33386129 10.1016/j.surg.2020.11.022

[53] Seykora TF et al (2019) The beneficial effects of minimizing blood loss in pancreatoduodenectomy. Ann Surg 270(1):147–157 29489483 10.1097/SLA.0000000000002714

[54] Pratt WB, Callery MP, Vollmer CM Jr. (2008) Risk prediction for development of pancreatic fistula using the ISGPF classification scheme. World J Surg 32(3):419–428 18175170 10.1007/s00268-007-9388-5

[55] Molinari E (2007) Amylase value in drains after pancreatic resection as predictive factor of postoperative pancreatic fistula: results of a prospective study in 137 patients. Ann Surg 246(2):281–287 17667507 10.1097/SLA.0b013e3180caa42f PMC1933557

[56] Strasberg SM et al (2002) Prospective trial of a blood supply-based technique of pancreaticojejunostomy: effect on anastomotic failure in the whipple procedure. J Am Coll Surg, 194(6): p. 746 – 58; discussion 759 – 60. 10.1016/s1072-7515(02)01202-4 12081065

[57] Connor S (2016) Defining post-operative pancreatitis as a new pancreatic specific complication following pancreatic resection. HPB (Oxford) 18(8):642–651 27485058 10.1016/j.hpb.2016.05.006 PMC4972444

[58] Bannone E et al (2021) Postoperative hyperamylasemia (POH) and acute pancreatitis after pancreatoduodenectomy (POAP): state of the art and systematic review. Surgery 169(2):377–387 32641279 10.1016/j.surg.2020.04.062

[59] Bannone E et al (2022) Postoperative serum hyperamylasemia (POH) predicts additional morbidity after pancreatoduodenectomy: it is not all about pancreatic fistula. Pancreatology 22(Supplement 1):e85 10.1016/j.surg.2022.04.003 35636983

[60] Heo D et al (2025) Clinical implications of postoperative hyperamylasemia and postpancreatectomy acute pancreatitis after pancreatectomy: a systematic review and meta-analysis. Surgery 184:109443 40435914 10.1016/j.surg.2025.109443

[61] Rystedt J et al (2019) Major intraoperative bleeding during pancreatoduodenectomy - preoperative biliary drainage is the only modifiable risk factor. HPB (Oxford) 21(3):268–274 30170978 10.1016/j.hpb.2018.07.024

[62] Nanashima A et al (2013) Predictive parameters of intraoperative blood loss in patients who underwent pancreatectomy. Hepatogastroenterology 60(125):1217–1221 23803385 10.5754/hge11376

[63] Uchiyama H et al (2015) Pancreatic transection using tape sling and ultrasonic aspirator dissection technique in pancreaticoduodenectomy and distal pancreatectomy. J Am Coll Surg 221(5):e91–e95 26329618 10.1016/j.jamcollsurg.2015.08.005

[64] Eng OS (2013) Safety and efficacy of LigaSure usage in pancreaticoduodenectomy. HPB (Oxford) 15(10):747–752 23782268 10.1111/hpb.12116 PMC3791113

[65] Gehrig T (2011) LigaSure versus conventional dissection technique in pancreatoduodenectomy: a pilot study. Am J Surg 201(2):166–170 20864081 10.1016/j.amjsurg.2010.02.023

[66] Ironside N (2018) Meta-analysis of an artery-first approach versus standard pancreatoduodenectomy on perioperative outcomes and survival. Br J Surg 105(6):628–636 29652079 10.1002/bjs.10832

