# Correction to: Clinical relevance of intraoperative blood loss in pancreatic surgery: a systematic review and meta-analysis to reappraise the impact on post operative pancreatic fistula

**DOI:** 10.1007/s00423-025-03938-8

**Published:** 2025-12-11

**Authors:** Giampaolo Perri, Danhui Heo, Rayner Peyser Cardoso, Swizel Ann Cardoso, Antonio Facciorusso, Riccardo Pellegrini, Domenico Bassi, Umberto Cillo, Giovanni Marchegiani

**Affiliations:** 1 Hepato Pancreato Biliary (HPB) and Liver Transplant Surgery, Department of Surgical, Oncological and Gastroenterological Sciences (DiSCOG), University of Padua, Via Giustiniani 2, Padova, 35128 Italy; 2 Doctoral School of Experimental and Preventive Medicine, Albert Szent-Györgyi Medical School, University of Szeged, Szeged, Hungary; 3 All India Institute of Medical Sciences, Jodhpur, India; 4 University Hospital of Birmingham NHS Foundation Trust, Birmingham, UK; 5 Gastroenterology Unit, Department of Experimental Medicine, Università del Salento, Lecce, 73100 Italy

**Correction to: Langenbeck’s Archives of Surgery (2025) 411:9**

10.1007/s00423-025-03902-6

The original version of this article contained an error. Table 2 was inadvertently omitted and is now provided below.

Also, the following sentence should have been added as the first sentence in the section “Association between intraoperative blood loss and POPF”: The characteristics of the 12 studies included in the quantitative synthesis are summarized in Table 2.

**Table 2:** Characteristics of included studies in the meta-analysis

Study	Design	Country	Study period	POPF definition	Group	Patients number	Age, y	Sex (Female)	Texture (Soft)	Duct size, mm	Pathology
Casciani et al.^*^ [47]	Retrospective	International	2003-2020	ISGPS	POPF	14.5% (N=1114)	N/A	N/A	85.6% (N=798)	≥ 3: 56.8% (N=529) < 3: 43.2% (N=403)	(Pancreatic cancer or pancreatitis): 32.1% (N=299)
					no POPF	85.5% (N=6592)	N/A	N/A	48.0% (N=2335)	≥ 3: 76.6% (N=3723) < 3: 23.4% (N=1140)	(Pancreatic cancer or pancreatitis): 43.7% (N=2123)
Cai et al. [50]	Restrospective	China	2019-2023	ISGPS	POPF	19.8% (N=65)	61.4 ± 4.5^§^	46.2% (N=30)	73.9% (N=48)	2.0 (1.3-2.6)^†^	N/A
					no POPF	80.2% (N=264)	65.7 ± 3.9^§^	40.9% (N=108)	29.2% (N=77)	2.3 (1.5-2.9)^†^	N/A
Dai et al. [49]	Retrospective	China	2014-2023	ISGPS	POPF	57.8% (N=52)	65.8 ± 10.9^‡^	11.5% (N=6)	64.1% (N=25)	3.0 ± 0.1^‡^	(Malignant): 84.6% (N=44)
					no POPF	42.2% (N=38)	63.1 ± 9.9^‡^	26.3% (N=10)	27.3% (N=6)	3.6 ± 0.2^‡^	(Malignant): 92.1% (N=35)
Furukawa et al. [14]	Retrospective	Japan	2014-2020	ISGPF	POPF	30.7% (N=23)	71 (62-74)^†^	21.7% (N=5)	87.0% (N=20)	2 (2-4)^†^	(Malignant): 13.0% (N=3)
					no POPF	69.3% (N=52)	68 (63-77)^†^	36.5% (N=19)	55.8% (N=29)	2 (2-2)^†^	(Malignant): 44.2% (N=3)
Hwang et al. [44]	Retrospective	Korea	2002-2008	ISGPF	POPF	17.0% (N=27)	62.9 ± 11.2^§^	N/A	N/A	3 (2-3)^†^	N/A
					no POPF	83.0% (N=132)	59.6 ± 12.0^§^	N/A	N/A	3 (2-5)^†^	N/A
Kawasaki et al. [36]	Retrospective	Korea	2006-2016	ISGPF	POPF	16.4% (N=10)	36 (24-80)	70.0% (N=7)	70.0% (N=7)	N/A	N/A
					no POPF	83.6% (N=51)	52 (21-86)	66.7% (N=34)	81.6% (N=40)	N/A	N/A
Kumar et al. [28]	Retrospective	India	2015-2017	ISGPF	POPF	20.9% (N=14)	48.2 ± 12.1^‡^	57.1% (N=8)	71.4% (N=10)	3.5 ± 2.6^‡^	(Malignant): 100% (N=14)
					no POPF	79.1% (N=53)	48.2 ± 11.3^‡^	39.6% (N=21)	46.5% (N=20)	4.3 ± 2.9^‡^	(Malignant): 100% (N=53)
Li et al. [34]	Retrospective	China	2011-2016	ISGPS	POPF	20.5% (N=61)	62.2 ± 9.4^§^	32.8% (N=20)	82.0% (N=50)	2.6 ± 0.7^§^	(Malignant): 91.8% (N=56)
					no POPF	79.5% (N=237)	61.8 ± 10.1^§^	43.0% (N=102)	33.8% (N=80)	3.2 ± 1.0^§^	(Malignant): 94.5% (N=224)
Marchegiani et al. [48]	Prospective	Italy	2016-2021	ISGPS	POPF	20.1% (N=182)	N/A	N/A	N/A	N/A	N/A
					no POPF	79.9% (N=722)	N/A	N/A	N/A	N/A	N/A
Sushma et al. [31]	Prospective	India	2019-2020	ISGPS	POPF	14.0% (N=7)	48 ± 13^§^	N/A	85.7% (N=6)	4 (3.0-4.5)^†^	N/A
					no POPF	86.0% (N=43)	54 ± 15^§^	N/A	32.6% (N=14)	4 (3.0-7.0)^†^	N/A
Tomimaru et al. [33]	Retrospective	Japan	2009-2017	ISGPS	POPF	24.1% (N=38)	70 ± 7^§^	26.3% (N=10)	97.4% (N=37)	2.8 ± 1.1^§^	(Malignant): 57.9% (N=22)
					no POPF	75.9% (N=120)	70 ± 10^§^	45.8% (N=55)	43.3% (N=52)	4.5 ± 2.0^§^	(Malignant): 77.5% (N=93)
Yang et al. [41]	Retrospective	China	2010-2013	ISGPF	POPF	16.2% (N=18)	56.6 ± 9.8^§^	44.4% (N=8)	83.3% (N=15)	≥ 3: 88.9% (N=16) < 3: 11.1% (N=2)	(Malignant): 83.3% (N=15)
					no POPF	83.8% (N=93)	56.4 ± 10.3^§^	51.6% (N=48)	75.3% (N=70)	≥ 3: 61.3% (N=57) < 3: 38.7% (N=36)	(Malignant): 87.1% (N=81)

* This study, total population (7706) and patient characteristic population (5795) are different. † Median, (IQR) is used. ‡ Median, CI 95% is used. § Mean, SD is used. Duct size refers to the main pancreatic duct (MPD). POPF: postoperative pancreatic fistula. ISGPS: international study group on pancreatic surgery. ISGPF: international study group of pancreatic fistula

The original article has been corrected.

