# Supplementary material for: Clinical relevance of intraoperative blood loss in pancreatic surgery: a systematic review and meta-analysis to reappraise the impact on post operative pancreatic fistula
Source: Langenbecks Arch Surg. 2025 Nov 10;411(1):9. doi: 10.1007/s00423-025-03902-6 (PMC12602675; doi:10.1007/s00423-025-03902-6)
Supplement: Supplementary file 2 — Supplementary Material 2 [file 423_2025_3902_MOESM2_ESM.docx]

**Supplementary Information**

**Supplementary Figure 1**

**Literature search**

Database:

**PubMed**

**((pancreaticoduodenectomy[Title/Abstract]) OR (pancreatectomy[Title/Abstract]) OR (pancreatoduodenectomy[Title/Abstract]) OR (duodenopancreatectomy[Title/Abstract]) OR (Whipple[Title/Abstract]) OR (Kausch-Whipple[Title/Abstract]) OR (PPPD[Title/Abstract]) OR (pancreatic resection[Title/Abstract]) OR (duodenum- preserving pancreatic head resection[Title/Abstract]) OR (hepatectomy[Title/Abstract]) OR (hepatic resection[Title/Abstract]) OR (liver resection[Title/Abstract])) AND ((intraoperative[Title/Abstract]) OR (intra-operative[Title/Abstract])) AND (blood[Title/Abstract]) AND (loss[Title/Abstract])**

**Supplementary Figure 2**

**
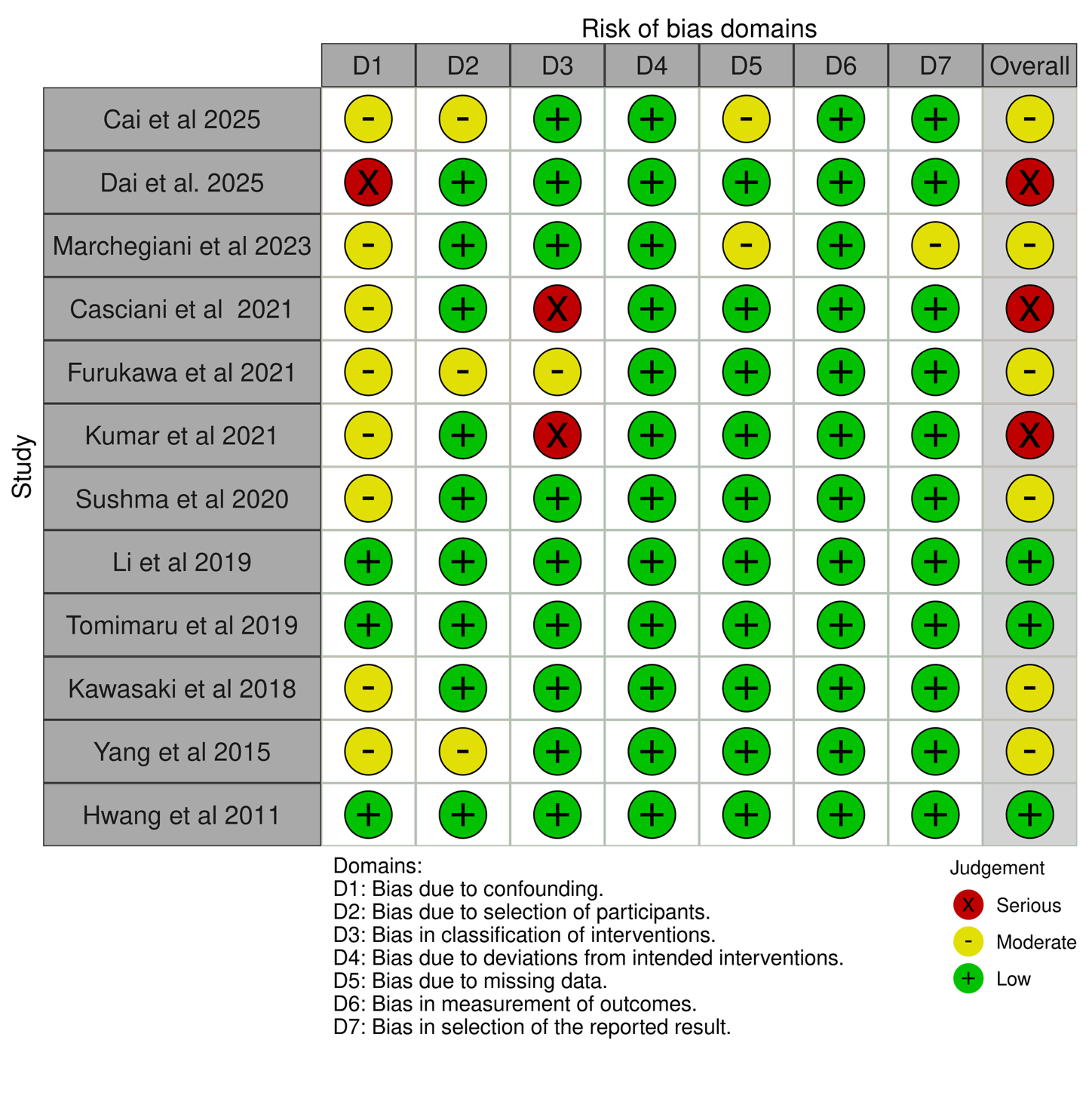

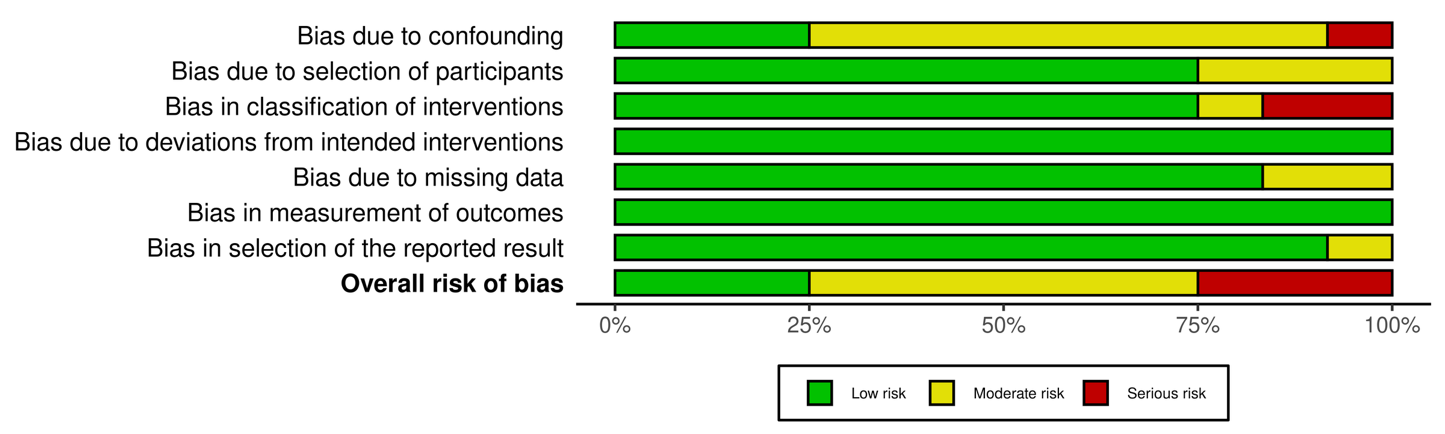
**

**Supplementary Figure 3**

**
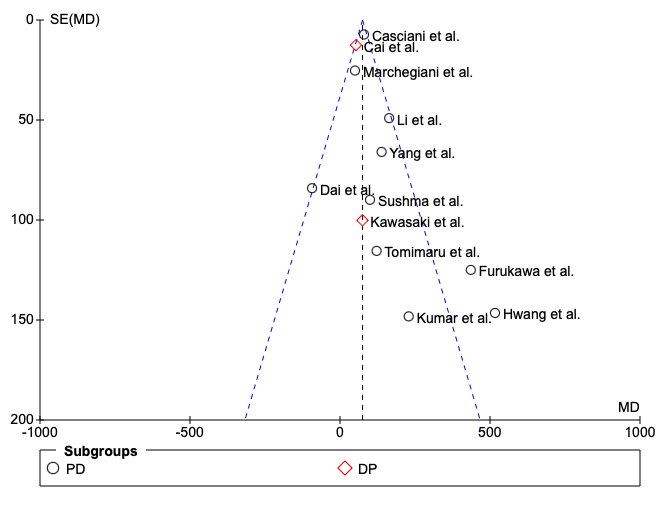
**

**Supplementary Figure 4**

**Author(s): Giampaolo Perri, Danhui Heo, Rayner Peyser Cardoso, Swizel Ann Cardoso, Antonio Facciorusso, Riccardo Pellegrini, Domenico Bassi, Umberto Cillo, and Giovanni Marchegiani**

**Question:** POPF compared to no POPF for health problem or population

**Setting:**

**Bibliography:**

| **Certainty assessment** | | | | | | | **№ of patients** | | **Effect** | | **Certainty** | **Importance** |
| --- | --- | --- | --- | --- | --- | --- | --- | --- | --- | --- | --- | --- |
| **№ of studies** | **Study design** | **Risk of bias** | **Inconsistency** | **Indirectness** | **Imprecision** | **Other considerations** | **POPF** | **no POPF** | **Relative (95% CI)** | **Absolute (95% CI)** |  |  |
| **intra- op bleeding (Subgroup PD)** | | | | | | | | | | | | |
| 10 | non-randomised studies | serious | very serious^a^ | not serious | not serious | publication bias strongly suspected^b^ | 1536 | 8082 | - | MD **112.46 higher** (30.39 higher to 194.53 higher) | ⨁◯◯◯ Very low^a,b^ |  |
| **Intra-op Bleeding (Subgroup DP)** | | | | | | | | | | | | |
| 2 | non-randomised studies | not serious | not serious | not serious | not serious | none | 75 | 315 | - | MD **53.67 ml higher** (19.68 higher to 87.65 higher) | ⨁⨁◯◯ Low |  |
| **Intra op bleeding Complete group** | | | | | | | | | | | | |
| 12 | non-randomised studies | serious^c^ | serious^d^ | not serious | not serious | publication bias strongly suspected^b^ | 1611 | 8397 | - | MD **112.46 ml higher** (30.39 higher to 164.53 higher) | ⨁◯◯◯ Very low^b,c,d^ |  |

**CI:** confidence interval; **MD:** mean difference

#### Explanations

a. We examined the heterogeneity and found substantial heterogeneity in point estimates across these eight studies. Because of that, we downgraded by 1 level for inconsistency.

b. asymmetric funnel

c. several serious ROBINS domains

d. I²=88%; subgroup p=0.08, I²=66.4
